# Fermentation Supernatants of *Pleurotus eryngii* Mushroom Ameliorate Intestinal Epithelial Barrier Dysfunction in Lipopolysaccharide-Induced Caco-2 Cells via Upregulation of Tight Junctions

**DOI:** 10.3390/microorganisms9102071

**Published:** 2021-10-01

**Authors:** Georgia Saxami, Evangelia N. Kerezoudi, Evdokia K. Mitsou, Georgios Koutrotsios, Georgios I. Zervakis, Vasiliki Pletsa, Adamantini Kyriacou

**Affiliations:** 1 Department of Nutrition and Dietetics, Harokopio University, 17671 Athens, Greece; gsaxami@hua.gr (G.S.); or evangelia.kerezoudi@oru.se (E.N.K.); emitsou@hua.gr (E.K.M.); 2 School of Medical Sciences, Örebro University, SE-701 82 Örebro, Sweden; 3 Laboratory of General and Agricultural Microbiology, Department of Crop Science, Agricultural University of Athens, 11855 Athens, Greece; georgioskoutrotsios@gmail.com (G.K.); zervakis@aua.gr (G.I.Z.); 4 Institute of Chemical Biology, National Hellenic Research Foundation, 11635 Athens, Greece; vpletsa@eie.gr

**Keywords:** *Pleurotus eryngii*, fermentation supernatants, *zonulin 1*, *occludin*, *claudin-1*, gut barrier

## Abstract

In recent years, modulation of gut microbiota through prebiotics has garnered interest as a potential to ameliorate intestinal barrier dysfunction. The aim of the study was to examine the in vitro effect of fermentation supernatants (FSs) from rich in β-glucan *Pleurotus eryngii* mushrooms on the expression levels of tight junctions (TJs) genes in Caco-2 cells stimulated by bacterial lipopolysaccharides (LPS). Mushrooms were fermented using fecal inocula in an in vitro batch culture model. Caco-2 cells were subjected to LPS and FS treatment under three different conditions: pre-incubation with FS, co- and post-incubation. Reverse transcription PCR was applied to measure the expression levels of *zonulin-1*, *occludin* and *claudin-1* genes. FSs from *P. eryngii* mushrooms led to a significant upregulation of the TJs gene expression in pre-incubation state, indicating potential preventive action. Down-regulation of all TJs gene expression levels was observed when the cells were challenged with LPS. The FS negative control (gut microbiota of each donor with no carbohydrate source) exhibited a significant upregulation of TJs expression levels compared to the cells that were challenged with LPS, for all three conditions. Overall, our data highlighted the positive and potential protective effects of *P. eryngii* mushrooms in upregulation of TJs’ genes.

## 1. Introduction

The intestinal barrier constitutes a physical barrier that separates the host’s mucosa milieu from the intestinal lumen. This barrier serves as a semipermeable structure that allows the absorption of nutrients and fluids but, simultaneously, prevents the transport of potentially harmful substances, such as antigens and microorganisms, from entering the underlying tissue [1,2]. The intestinal barrier consists of four layers and includes [3,4]: (1) intestinal alkaline phosphatase, released by intestinal epithelial cells which dephosphorylates lumimal lipopolysaccharides (LPS); (2) the mucus layer that provides the first physical barrier preventing interactions between gut microbiota and intestinal cells; (3) a single cell layer, consisting of various cell types, such as enterocytes, goblet and Paneth cells, which separates the lumen from the systemic circulation and (4) antibacterial proteins secreted by Paneth cells along with IgA secretion from plasma cells present in the lamina propria.

The space between epithelial cells in the intestinal barrier is regulated through the presence of junctional complexes including desmosomes, adherens junctions and tight junctions (TJs) [5]. TJs are located at the apical portion of the lateral membrane of intestinal epithelial cells, sealing the intercellular space and regulate paracellular permeability across the epithelium [6]. TJs are protein complexes that include transmembrane proteins, which are embedded into the plasma membrane and interact with proteins from adjacent cells, including claudins, three junctional MARVEL domain containing proteins (MAL and related proteins for vesicle trafficking and membrane link, i.e., occludin, tricellulin and MARVELD3) and members of the cortical thymocyte marker in *Xenopus* family of adhesion molecules, such as junctional adhesion molecules (JAMs) [7,8]. TJs also contain a cytoplasmatic plaque that connects the transmembrane proteins to the actinomyosin cytoskeleton of the cell, where zonula occludens proteins (ZO-1, ZO-2 and ZO-3) and the two mammalian polarity complexes PAR-3 (partition defective)/PAR-6/aPKC (atypical protein kinase C) and Crumbs/PALS1 (proteins associated with Lin seven)/PATJ (protein-associated with TJ) are predominant [9]. Disrupted TJs lead to increased gut permeability, a condition referred to as ‘leaky gut’, resulting to the uncontrolled translocation of pathogens, antigens and toxins from the lumen to lamina propria into the blood stream. Leaky gut is associated with the development of various diseases such as inflammatory bowel diseases (IBD), irritable bowel syndrome (IBS) and obesity [10,11,12].

Modulation of gut microbiota through probiotics and prebiotics for reversing the state of leaky gut has garnered considerable interest in recent years. Prebiotics are defined as “nondigestible food ingredients that when administered, exert a beneficial effect on the host health” [13]. The most recognized prebiotics in human diet are fructo-oligosaccharides (FOS), galacto-oligosaccharides (GOS), lactulose and inulin, whereas β-glucans derived from various mushroom species (i.e., *Pleurotus ostreatus* and *Pleurotus eryngii*) are potential prebiotic candidates [14,15]. *P. eryngii* is an edible mushroom, widely consumed in many countries worldwide due to its nutritional value and biological functions, and several studies have revealed a multitude of beneficial effects such as immunostimulatory, anti-tumor, antioxidant, antimicrobial and antiviral activities [16,17,18,19,20]. Several studies have confirmed the ability of probiotics or their surface components and metabolites to reverse the leaky gut state via regulating TJs [21,22,23,24]. On the other hand, there are fewer studies that examine the effect of prebiotics on the restoration of the intestinal barrier by regulating the TJs [25,26]. However, most of the studies conducted with prebiotics do not take into account the fermentation process taking place in the gut and the possible synergistic activities among metabolites. There are only few studies available examining the effect of fermentation supernatants (FSs) from prebiotic substances on the intestinal barrier integrity [2,27,28,29].

The aim of the present study was to investigate the impact of FSs from rich in β-glucans *P. eryngii* edible mushrooms on the expression levels of TJs’ genes in LPS-stimulated Caco-2 cells using three different conditions, i.e., pre-treatment with FS, co-incubation with FS and LPS treatment, as well as post-treatment with FS of Caco-2 cells subjected initially to LPS challenge.

## 2. Materials and Methods

### 2.1. Fermentation Supernatants (FSs) Preparation

In vitro static batch cultures inoculated with fecal samples from eight apparently healthy volunteers over 65 years old were performed for 24 h in the presence of lyophilised powder from mushrooms of *P. eryngii* strain LGAM 216, as previously described [14]. Basal fermentation medium had no cytotoxic effect, as previously described [14]. For the collection of the FSs, samples were harvested from the in vitro fermentation at 24 h, followed by centrifugation at 10,000× *g* for 30 min at 4 °C. The supernatants were collected and filtered through a 0.22-μm pore filter (Millex^®^, Merck KGaA, Darmstadt, Germany), and stored at −20 °C. The culture medium of the static batch culture, without any carbon source but inoculated with gut microbiota of each donor, was used as negative control, as mentioned in the Table 1.

### 2.2. Bacterial Lipopolysaccharides (LPS)

Bacterial LPS isolated from *Escherichia coli* O55: B5 (Cayman Chemical Company, Ann Arbor MI, USA) were used for the impairment of the intestinal barrier permeability.

### 2.3. Cell Lines

The Caco-2 cell line has been derived from human colon adenocarcinoma. Caco-2 (Caco2) cells (ATCC^®^-HTB-37^TM^) were cultured on Dulbecco’s Modified Eagle Medium (DMEM) medium supplemented with stable glutamine (Biosera, Nuaille, France), enriched with 1% penicillin/streptomycin (10,000 U/mL, Biochrom AG, Berlin, Germany) and 10% (*v/v*) fetal bovine serum (Biochrom AG), at 37 °C, 95% humidity and 5% CO_2_. Cells from passages 20 to 30 were used for all experiments.

### 2.4. Cell Viability Assay

Viability of Caco-2 cells, challenged with LPS or FSs, was assessed by the 3-(4,5-dimethyl thiazol-2-yl)-2,5-diphenyl tetrazolium bromide (MTT) assay [30]. Briefly, Caco-2 cells were cultured in DMEM medium, placed on a 96-well plate and stabilized for 24 h. Then, cells were treated with different concentrations of LPS (80, 100, 120, 150 and 200 ng/mL) for 3, 24, 48 and 72 h. FSs (FS-PEWS, FS-PEWSGM and FS-NC) were diluted with DMEM culture medium at concentrations 1, 2, 5, 8 and 10% (*v/v*) and were added to the cells for 3, 24, 48 and 72 h. After treatment with the selected concentrations, cells were incubated with 15 μL of filtered MTT solution (5 mg/mL) (Life Technologies Co., Carlsbad, CA, USA), diluted in pre-warmed PBS, for 4 h at 37 °C. Then, the medium was removed and 100 μL of dimethyl sulfoxide (AppliChem GmbH, Darmstadt, Germany) were added to each well for dissolving the formazan product from cells. Finally, the absorbance was measured at 540 nm using a PowerWave™ XS microplate reader (BioTek Instruments Inc., Winooski, VT, USA), while the collection of results was performed using the Gen5 software (BioTek Instruments Inc., Winooski, VT, USA). Cell viability was expressed as the % of untreated cells. All experiments were performed in triplicate wells for each condition and repeated at least twice.

### 2.5. Challenge of Caco-2 Cells with FSs and LPS

Caco-2 cells were seeded in six-well plates in complete medium at a density of 135,000 cells/well for 48 h or 130,000 cells/well for 72 h of incubation at 37 °C in 5% CO_2_. Then, Caco-2 cells were subjected to LPS and FS treatment under three different conditions:

(1) Pre-incubation (FS/LPS): incubation of the Caco-2 cell line with FSs of PEWS or PEWSGM mushrooms for 48 h and then exposure to LPS for 24 h (protective effect),
(2) Co-incubation (FS + LPS): simultaneous incubation of the Caco-2 cell line with the FSs of PEWS or PEWSGM mushrooms and the LPS for 48 h (protective effect),
(3) Post-incubation (LPS/FS): exposure of the Caco-2 cell line to LPS for 24 h and then incubation with the FSs of PEWS or PEWSGM mushrooms for 48 h (reparative effect).

Additionally, cells treated with LPS (Cells + LPS) for impairing intestinal barrier were designated as the positive control.

### 2.6. RNA Extraction and cDNA Synthesis

Total RNA was isolated with NucleoZOL reagent (MACHEREY-NAGEL GmbH & Co. KG, Dueren, Germany) according to manufacturer instructions. The quality and quantity of the isolated RNA were determined spectrophotometrically and by agarose gel electrophoresis. One microgram of total RNA was reverse transcribed into cDNA using PrimeScript First Strand cDNA Synthesis Kit (Takara Bio Inc., Kusatsu, Shiga, Japan).

### 2.7. Quantitative Real-Time PCR

Quantitative real-time PCR was performed on a StepOne PCR System in MicroAmp^®^ Fast Optical 48-Well Reaction Plates (both from Thermo Fisher Scientific, Waltham, MA, USA) using the KAPA SYBR^®^ FAST qPCR Kit (Kapa Biosystems, Wilmington, MA, USA) under the following conditions: 95 °C for 3 min followed by 40 cycles of 95 °C for 15 s and 60 °C for 1 min. The housekeeping gene *b-actin* was used as an internal control for normalization, while untreated cells were used as reference sample. Each reaction was performed in duplicate. The sequences of TJ primers are presented in Table 2. Primer specificity was verified by performing a melting curve analysis. For relative quantification of the transcripts, the formula RQ = 2^−ΔΔCt^ was used.

### 2.8. Statistical Analysis

Normality of the data was assessed using the Shapiro–Wilk test and by visualizing the data in histograms. Continuous variables are presented as mean value ± standard deviation (SD). The pair-wise comparisons within same groups (e.g., treatments) were performed using Wilcoxon matched-pairs signed rank test on non-parametric data and paired samples *t*-test on parametric data. The software program IBM^®^ SPSS^®^ Statistics version 21 was used for the statistical analysis of the results and the significance threshold was set at 5% (*p* < 0.05).

## 3. Results

### 3.1. Effect of FSs and LPS on Cell Viability in Caco-2 Cells

**Figure 1:**
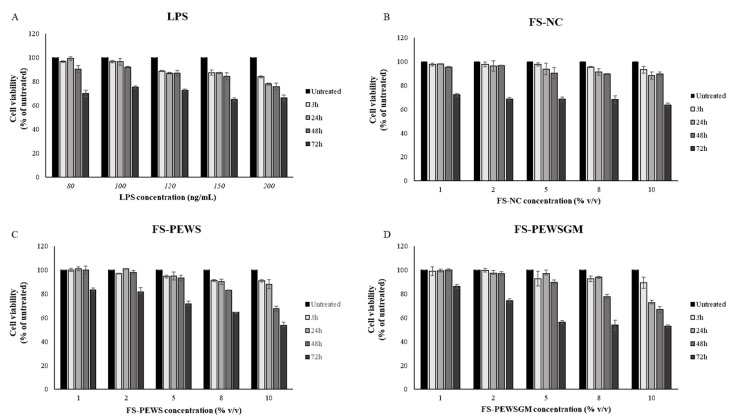
Effects of lipopolysaccharides (LPS) and FSs (FS-NC, FS-PEWS and FS-PEWSGM) on Caco-2 cell viability. Caco-2 cells were seeded in a 6-well plate and treated with (**A**) LPS at concentrations of 80, 100, 120, 150 and 200 ng/mL for 3, 24, 48 and 72 h, (**B**) FS-NC at concentrations of 1%, 2%, 5%, 8% and 10% for 3, 24, 48 and 72 h, (**C**) FS-PEWS at concentrations of 1%, 2%, 5%, 8% and 10% for 3, 24, 48 and 72 h, and (**D**) FS-PEWSGM at concentrations of 1%, 2%, 5%, 8% and 10% for 3, 24, 48 and 72 h. Values are mean ± SD of three independent experiments.

MTT assay was performed for the determination of optimal concentrations of FSs and LPS, as well as for the determination of the optimal incubation time in order to challenge Caco-2 cells. The viability of Caco-2 cells was examined after LPS challenge for 3, 24, 48, and 72 h at concentrations of 80, 100, 120, 150 and 200 ng/mL, as shown in Figure 1A. Similarly, the viability of Caco-2 cells after incubation with 1, 2, 5, 8 and 10% (*v/v*) of FSs (FS-PEWS, FS-PEWSGM and FS-NC) for 3, 24, 48, and 72 h was examined (Figure 1B,C). As shown in Figure 1A, LPS treatment induced a significant reduction in cell viability of Caco-2 cells, at concentrations of 120, 150 and 200 ng/mL, for all time points in a time- and dose-dependent manner. The incubation of Caco-2 cells with 80 ng/mL of LPS led to cell viability higher than 95% after 3h (96.76% ± 0.55) and 24 h (99.37% ± 1.47) of incubation, whilst cell viability was significantly reduced after 48 h (90.39% ± 2.99) and 72 h (70.04% ± 2.59). Finally, the incubation of Caco-2 cells with 100 ng/mL of LPS led to a decreased viability in a time- and dose-dependent manner, while after 24 h (96.67% ± 2.87) of incubation survival was over 95%. In conclusion, the optimal concentration for LPS was 100 ng/mL for 24 h of incubation.

Cell viability was practically unaffected after the incubation of Caco-2 cells with 1% and 2% of FS-NC for 3, 24 and 48 h as compared to untreated cells, with all cell survival rates remaining above 95% (Figure 1B). Incubation of Caco-2 cells with higher concentrations (5, 8 and 10%) for 24 and 48 h resulted in a significant reduction in cell viability (<95%) in a time- and dose-dependent manner. Finally, all subtest concentrations of FS-NC led to a significant reduction in cell viability at 72 h of incubation. Based on the cellular toxicity assay, a concentration of 2% of FS-NC for 48 h of incubation was used for the rest of the experiments (96.62% ± 0.30) (Figure 1B).

Similarly with the FS-NC, incubation of Caco-2 cells for 3, 24 and 48 h with FS-PEWS (Figure 1C) and FS-PEWSGM (Figure 1D), for concentrations of 1% and 2%, preserved the cell viability at levels higher than 95% compared to the untreated cells. In contrast, incubation of cells with both FSs (FS-PEWS or FS-PEWSGM) for 72 h for all the examined concentrations resulted in a significant reduction in cell viability in a dose dependent manner. In particular, incubation of cells with the FSs of PEWS or PEWSGM for 72 h, at concentration of 10%, led to a reduction in viability by 46.4% and 47.07%, respectively (Figure 1C). Incubation of Caco-2 cells with 2% of FS-PEWS or FS-PEWSGM, for 48 h led to viability values of 97.95% ± 1.7 and 96.90% ± 1.44, respectively. Thus, 2% of FS-PEWS and FS-PEWSGM were chosen as the optimal concentrations for further experiments. Finally, 48 h were found to be the optimal incubation time in order to challenge Caco-2 cells with the fermentation products.

### 3.2. Effect of FS-PEWS and FS-PEWSGM on Zonulin-1 Expression Levels

**Figure 2:**
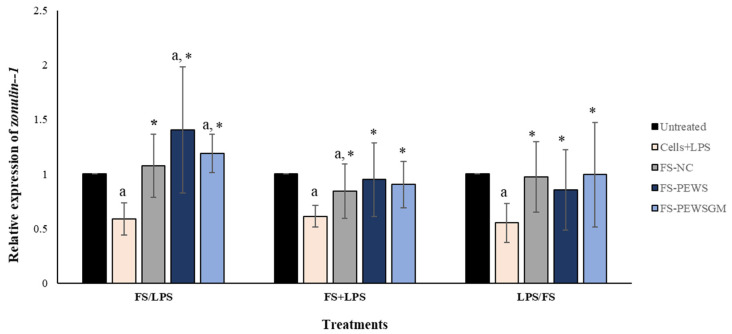
The effects of FSs from *P. eryngii* mushrooms (FS-PEWS and FS-PEWSGM) on *zonulin-1* expression levels in LPS-induced Caco-2 cells. Caco-2 cells were subjected to LPS and FS treatment under three different conditions: pre incubation (FS/LPS), co-incubation (FS + LPS) and post-incubation (LPS/FS). Untreated: Caco-2 cells without any effect; FS-NC: FS of the negative control (NC: sample without additional carbon source); Cells + LPS: Caco-2 cells subjected to LPS (100 ng/mL). Data are expressed as mRNA expression (normalized with *b-actin*) relative to untreated cells as mean ± SD of two independent experiments. ^a^ statistically significant for FS-PEWS or FS-PEWSGM compared to untreated cells (*p* < 0.05), * statistically significant for FS-PEWS or FS-PEWSGM compared to Cells + LPS (*p* < 0.05) (Wilcoxon matched-pairs signed rank test or paired samples *t*-test).

In order to evaluate the potential beneficial effects of FSs on intestinal epithelial barrier function disrupted by LPS in Caco-2 cell monolayers, the expression levels of TJs involved in the formation, function and integrity of the intestinal barrier were measured. Figure 2 depicts the results for the total of eight volunteers regarding the expression levels of the TJ *zonulin-1*, after incubation of the Caco-2 cells with FS-PEWS or FS-PEWSGM, under three different conditions: pre-incubation (FS/LPS), co-incubation (FS + LPS) and post-incubation (LPS/FS). As previously mentioned, cells incubated only with LPS (cells + LPS) were also used to confirm intestinal barrier disassembly. As shown in Figure 2, incubation of Caco-2 cells with LPS resulted in a significant reduction in *zonulin-1* gene expression levels for all three conditions compared to the untreated cells (FS/LPS: 0.588 ± 0.147, FS + LPS: 0.613 ± 0.099, LPS/FS: 0.553 ± 0.177, *p* for all <0.001). In the pre-incubation state, the treatment of Caco-2 cells with the mushroom FSs (FS-PEWS and FS-PEWSGM) led to a significant increase in the expression levels of *zonulin-1* compared to the untreated cells (FS-PEWS: 1.403 ± 0.579; FS-PEWSGM: 1.188 ± 0.177, *p* for all <0.001) and the cells that were subjected to LPS (FS-PEWS vs. Cells + LPS: 1.403 ± 0.579 vs. 0.588 ± 0.147; FS-PEWSGM vs. Cells+LPS: 1.188 ± 0.177 vs. 0.588 ± 0.147, *p* for all <0.001). Additionally, a positive trend was detected after pre-incubation of Caco-2 cells with FS-PEWS compared to FS-NC (1.075 ± 0.286, *p* = 0.063). In the same treatment, incubation of the cells with the FSs with only fecal inoculum and no carbon source during fermentation (FS-NC) resulted in upregulation of *zonulin-1* compared to cells that were subjected to LPS (FS-NC vs. Cells + LPS: 1.075 ± 0.289 vs. 0.588 ± 0.147, *p* < 0.001).

Regarding the co-incubation condition (Figure 2), when Caco-2 cells were subjected to simultaneous treatment (FS + LPS) with the FS-PEWS or FS-PEWSGM, the expression levels of *zonulin-1* gene were significantly upregulated to the extent of 0.950 ± 0.338 and 0.904 ± 0.212, respectively, as compared to cells that were challenged with LPS (*p* for all <0.001), while no significant difference was observed compared to the untreated cells. In fact, co-incubation of Caco-2 cells with FS-PEWSGM and LPS led to a decrease in the expression levels of *zonulin-1* gene, compared to the untreated cells (0.904 ± 0.212, *p* = 0.089). Furthermore, regarding incubation of Caco-2 cells with the FS-NC, *zonulin-1* gene exhibited a significant upregulation compared to cells that were challenged with LPS (FS-NC vs. Cells + LPS: 0.843 ± 0.251 vs. 0.613 ± 0.099, *p* < 0.001), while a significant reduction was observed compared to the untreated cells (*p* < 0.001).

Under the post-incubation condition (Figure 2), challenge of Caco-2 cells with FS-PEWS or FS-PEWSGM increased the relative expression levels of *zonulin-1* to the extent of 0.854 ± 0.338 (*p* < 0.05) and 0.904 ± 0.212 (*p* < 0.05), compared with cells that were challenged with LPS.

In conclusion, regarding the *zonulin-1* expression levels, incubation of the Caco-2 cells with FS-PEWS or FS-PEWSGM led to a significant increase only in the pre-incubation state, compared to untreated cells and cells that were challenged with LPS, while incubation with the FS of PEWS mushroom led to the highest mean value, which however did not differ significantly compared to FS-PEWSGM.

### 3.3. Effect of FS-PEWS and FS-PEWSGM on Occludin Expression Levels

**Figure 3:**
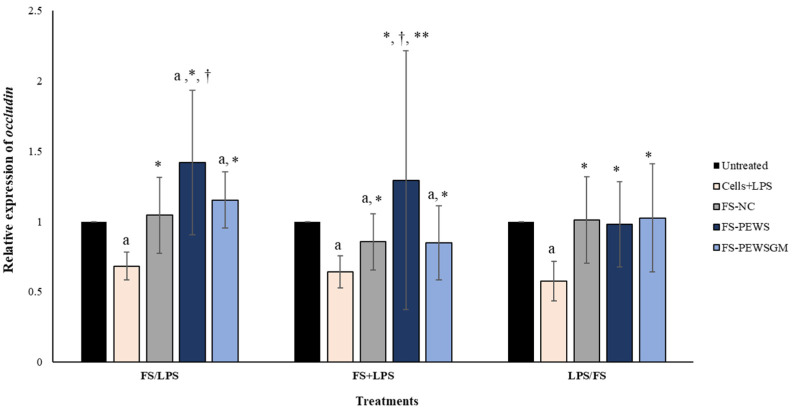
The effects of FSs from *P. eryngii* mushrooms (FS-PEWS and FS-PEWSGM) on *occludin* expression levels in LPS-induced Caco-2 cells. Caco-2 cells were subjected to LPS and FS treatment under three different conditions: pre incubation (FS/LPS), co-incubation (FS + LPS) and post-incubation (LPS/FS). Untreated: Caco-2 cells without any effect; FS-NC: FS of the negative control (NC: sample without additional carbon source); Cells + LPS: Caco-2 cells subjected to LPS (100 ng/mL). Data are expressed as mRNA expression (normalized with *b-actin*) relative to untreated cells as mean ± SD of two independent experiments. ^a^ statistically significant for FS-PEWS or FS-PEWSGM compared to untreated cells (*p* < 0.05), ^†^ statistically significant for FS-PEWS or FS-PEWSGM compared to FS-NC (*p* < 0.05), * statistically significant for FS-PEWS or FS-PEWSGM compared to Cells + LPS (*p* < 0.05); ** statistically significant for FS-PEWS compared to FS-PEWSGM (*p* < 0.05) (Wilcoxon matched-pairs signed rank test or paired samples *t*-test).

The relative expression of *occludin* in Caco-2 cells after incubation with FS-PEWS or FS-PEWSGM under different conditions (FS/LPS, FS + LPS, LPS/FS) is presented in Figure 3. In pre-incubation state, when Caco-2 cells were challenged with FS-PEWS the fold increase in *occludin* gene expression was 1.419 ± 0.513, compared to the untreated cells (*p* < 0.001), cells that were subjected to LPS (0.683 ± 0.099, *p* < 0.001) and FS-NC (1.045 ± 0.269, *p* < 0.001). The incubation of cells with FS-PEWSGM induced a significant increase in the gene expression levels of *occludin* (1.152 ± 0.200) compared to the untreated cells (*p* < 0.001) and cells that were challenged with LPS (0.683 ± 0.099, *p* < 0.001). On the other hand, in the co-incubation state, treatment of the Caco-2 cells with the FSs of both mushrooms led to an increment of *occludin* expression levels, without significant differences compared to the untreated cells (FS-PEWS: 1.293 ± 0.921; FS-PEWSGM: 0.849 ± 0.262, *p* for all >0.05). In the same condition, incubation of Caco-2 cells with FS-PEWS presented a notable upregulation of the expression levels of the *occludin* gene, which was significant in relation to cells that were challenged with LPS (0.642 ± 0.116, *p* < 0.001), FS-PEWSGM (0.849 ± 0.262, *p* < 0.001) and FS-NC (0.856 ± 0.199, *p* < 0.05). For the post incubation, a same pattern with the *zonulin-1* gene was observed, with the expression levels of the *occludin* gene being restored after incubation of Caco-2 cells with FS-PEWS, FS-PEWSGM and FS-NC (FS-PEWS: 0.981 ± 0.302 *p* < 0.001; FS-PEWSGM: 1.026 ± 0.384 *p* < 0.001; FS-NC: 1.011 ± 0.307 *p* < 0.001) compared to the cells that were subjected to LPS (Cells + LPS: 0.577 ± 0.141)

### 3.4. Effect of FS-PEWS and FS-PEWSGM on Claudin-1 Expression Levels

**Figure 4:**
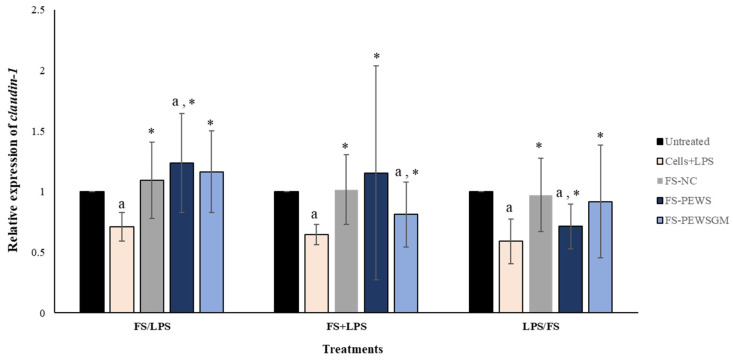
The effects of FSs from *P. eryngii* mushrooms (FS-PEWS and FS-PEWSGM) on *claudin-1* expression levels in LPS-induced Caco-2 cells. Caco-2 cells were subjected to LPS and FS treatment under three different conditions: pre incubation (FS/LPS), co-incubation (FS + LPS) and post-incubation (LPS/FS). Untreated: Caco-2 cells without any effect; FS-NC: FS of the negative control (NC: sample without additional carbon source); Cells + LPS: Caco-2 cells subjected to LPS (100 ng/mL). Data are expressed as mRNA expression (normalized with *b-actin*) relative to untreated cells as mean ± SD of two independent experiments. ^a^ statistically significant for FS-PEWS or FS-PEWSGM compared to untreated cells (*p* < 0.05), * statistically significant for FS-PEWS or FS-PEWSGM compared to Cells + LPS (*p* < 0.05) (Wilcoxon matched-pairs signed rank test or paired samples *t*-test).

The effects of FS-PEWS or FS-PEWSGM in Caco-2 cells under different conditions (FS/LPS, FS + LPS, LPS/FS) on *claudin-1* expression levels is demonstrated in Figure 4. In the pre-incubation state, treatment of Caco-2 cells with the FS-PEWS resulted in a significant increase of *claudin-1* (1.236 ± 0.409, *p* < 0.05), compared to the untreated cells and cells that were subjected to LPS. Incubation with FS-PEWSGM under the same condition displayed a significant increase (1.165 ± 0.336; *p*< 0.05) in the expression levels of *claudin-1* compared to the cells that were challenged with LPS (0.711 ± 0.119; *p*< 0.05); however, no significant difference to the untreated cells was detected. For the co-incubation treatment, incubation of Caco-2 cells with FS-PEWS led to a statistically significant increment (1.155 ± 0.882) compared to cells that were challenged with LPS (*p* < 0.05). Similarly, the addition of FS-PEWSGM resulted in a significant increase of *claudin-1* gene expression compared to cells that were subjected to LPS, while a significant decrease was observed in comparison to the untreated cells (0.812 ± 0.269, *p* < 0.05). Finally, concerning the post-incubation treatment, challenge of Caco-2 cells with FS-PEWS or FS-PEWSGM increased the relative expression levels of *claudin-1* transcripts compared with cells that were challenged with LPS. Incubation of Caco-2 cells with FS-NC for all 3 conditions led to a significant upregulation in the expression levels of the *claudin-1* gene compared to the cells that were subjected to LPS. Since similar observations were made for the other two genes examined (*zonulin-1* and *occludin*), a positive effect of the intestinal microbiota in the restoration of distributed TJs could be suggested.

### 3.5. Effect of FSs on TJs Expression Levels per Volunteer

Table 3 demonstrates the results of TJs expression levels, for all three conditions (pre-, co- and post-incubation) per volunteer. For most volunteers (volunteer 2, 3, 4, 6, 7 and 8), pre-incubation of Caco-2 cells with FSs of both mushrooms resulted in the highest expression levels of *zonulin-1* gene compared to the other two conditions (co- and post-incubation). In post-incubation, a decrease of *zonulin-1* expression levels was observed in most volunteers, while in donors 4 and 5, treatment of Caco-2 cells with FS-PEWS led to a significant decrease compared to the untreated cells (volunteer 4: FS-PEWS: 0.720 ± 0.028; volunteer 5: FS-PEWS: 0.310 ± 0.014, *p* for all <0.05). Additionally, except for volunteer 6, incubation of cells with FS-NC led to increased expression levels of *zonulin-1* gene in the pre-incubation condition, as compared to the other two conditions.

Pre-incubation of Caco-2 cells with FS-PEWS led to the highest expression levels of *occludin*, compared to the other two states, for most volunteers (volunteer 1, 2, 3, 4, 5, and 8). On the other hand, incubation of cells with FS-PEWSGM resulted to increased expression levels of *occludin*, only in the case of volunteer 2, 3 and 8, under the same treatment. In the case of incubation of cells with FS-NC, a same pattern with *zonulin-1* was observed. More specifically, pre-incubation of Caco-2 cells with FS-NC led to the highest expression levels of *occludin* in the vast majority of volunteers (volunteer 1, 2, 3, 4, 5, 7 and 8). In co-incubation state, treatment of Caco-2 cells with FS-PEWS led to higher expression levels of *occludin* gene compared to FS-PEWSGM for all volunteers. Furthermore, under the same condition, incubation of cells with FS-NC led to increased expression levels of *occludin* compared to FS-PEWSGM for most volunteers (volunteer 2, 3, 4, 5 and 8). In the post-incubation state, treatment of cells with FS-PEWS resulted in higher levels of *occludin* compared to FS-PEWSGM in half of the volunteers (volunteer 2, 3, 7 and 8).

Regarding *claudin-1*, pre-incubation of Caco-2 cells with FS-PEWS or FS-PEWSGM resulted in higher expression levels, as observed for most volunteers (volunteer 1, 2, 3, 4 and 8 for FS-PEWS and volunteers 2, 3, 5, 7 and 8 for FS-PEWSGM), compared to the co- and post-incubation states. In co-incubation state, treatment of Caco-2 cells with FS-PEWS led to increased levels of *claudin-1* gene compared to FS-PEWSGM for most volunteers (volunteer 3, 4, 5, 6 and 7). In post-incubation condition, treatment of Caco-2 cells with FS-PEWS led to augmented expression levels of *claudin-1* compared to FS-PEWSGM in half of the volunteers (volunteer 2, 3, 6 and 7). Finally, pre-incubation of cells with FS-NC led to higher expression levels of *claudin-1* gene, compared to co- and post-incubation condition, for most volunteers (volunteer 1, 2, 3, 6, 7 and 8 for co-incubation and volunteer 1, 2, 3, 5 and 7 for post-incubation).

## 4. Discussion

Intestinal barrier function is crucial for normal gut homeostasis, while dysregulation of this barrier is associated with abnormal increased intestinal permeability, which allows the permeation of harmful molecules and organisms. This situation encourages the development of various gastrointestinal and systemic diseases such as IBD, IBS, diabetes and multiple sclerosis [33,34]. Restoration of impaired intestinal barrier through dietary components, such as probiotics and prebiotics, has been the subject of intensive research in recent years [35,36,37]. So far, many in vitro and in vivo studies have focused on the effects of prebiotics on the intestinal barrier function by examining the oligosaccharides without considering the fermentation process taking place in the gut and the possible synergistic actions that may develop among metabolites [2,38]. In addition, the few available studies that examine the fermentation products have examined known prebiotic compounds, such as inulin and galactooligosaccharides [2,27].

In the present study, the effect of FSs of *P. eryngii* mushrooms (PEWS and PEWSGM) on the intestinal barrier function was investigated in vitro via the LPS-stimulated Caco-2 cell line. According to our previous publication [14], these edible mushrooms have been already examined in terms of their prebiotic potential on the gut microbiota composition and metabolites by using in vitro static batch culture fermentation and fecal inocula from eight healthy elderly donors. The results suggested that both mushrooms were rich in β-glucans and could enhance the presence of beneficial bacteria (*Lactobacillus* and *Bifidobacterium*), as well as the production of short-chain fatty acids. The aim of this work was to investigate the impact of FSs (FS-PEWS and FS-PEWSGM) on the expression levels of TJs’ genes in LPS-stimulated Caco-2 cells. According to our results, both types of FS from *P. eryngii* could strongly impact the TJs gene expression in the condition of pre-incubation, indicating potential preventive action.

Zonulin-1 is a intercellular scaffolding protein that links the transmembrane proteins to the cytoskeletal proteins, which contributes to the regulation of TJs and the paracellular porosity [39,40]. Herein, pre-incubation of Caco-2 cells with FSs of PEWS and PEWSGM mushrooms led to a significant increase in the expression levels of *zonulin-1* gene compared to the untreated cells and the cells that were subjected to LPS (Figure 2), indicating their possible protective effect against intestinal barrier impairment. In this respect, Uerlings et al. (2011) [2] has reported an upregulation of *zonulin-1*, *claudin-1* and *claudin-3* genes in IPEC-J2 cells supplemented with inulin FS, compared to control cells and digested inulin, suggesting that metabolites arising from the fermentation process could be responsible for the invigoration of the barrier function. Many studies have examined the effects of individual SCFAs in intestinal barrier function; however, the effects of mixed SCFAs or fermentation products on intestinal barrier function have been rarely investigated [29]. Recently, Yiu et al. (2021) [29], examined the effects of fermentation products of polydextrose, lactitol and xylitol on impaired colonic epithelial barrier function in vitro. The authors pointed out that fermentation of polydextrose, lactitol and xylitol produced different amounts of SCFAs and that fermentation of xylitol resulted in better reparative effects than polydextrose, suggesting that specific ingredients, which mainly produce butyrate during fermentation, may be of more value for improving gut health [23]. On the other hand, Peng et al. (2009) [41] noted that the treatment of Caco-2 cells with butyrate did not significantly alter the expression levels of TJ proteins (occludin, claudin-1, claudin-4, and ZO-1). Therefore, regulation of barrier function by butyrate in the Caco-2 cell model is unlikely to be explained by the changes in the expression of these proteins. For the other two treatments (co-incubation and post-incubation), there was a significant increase in *zonulin-1* expression levels after incubation of the Caco-2 cells with the FSs of both mushrooms only compared to the cells that were challenged with LPS, indicating a positive trend in the restoration of the intestinal barrier. Although there are no publications reporting on the effects of prebiotics in these three conditions, several studies suggest the efficacy of prebiotics in protection of intestinal barrier integrity. Ducray et al. (2019) [37] demonstrated that oral treatment of rats with *Saccharomyces cerevisiae* fermentate prebiotic (before exposure to heat stress conditions) maintained expression of TJ proteins (occludin, claudin, ZO-1 and JAM-A). In addition, in vivo and in vitro studies have shown that galactooligosaccharides pretreatment in LPS-challenged mice resulted in upregulation of *zonulin-1*, *occludin* and *claudin-1* gene expression [25,42]. Sheng et al. (2020) [43], showed that pre-administration of xyloligosaccharides for 21 days in C57BL/6 mice was able to protect intestinal barrier integrity by increasing the expression levels of TJs.

Occludin was the first identified TJ protein and together with claudins are the main transmembrane proteins that contribute to the paracellular seal by linking adjacent cells to the actin cytoskeleton through cytoplasmatic scaffolding proteins, such as zonula occludens [23,44,45]. The interaction between occludin and zonulin-1 or zonulin-2 protein is crucial for maintaining normal function of the intestinal barrier as well as the integrity of the TJs [44]. Occludin plays an important role in the maintenance and assembly of TJs, while many in vitro and in vivo studies have shown that the expression levels of occludin were downregulated in disturbed intestinal barrier [23,46,47]. In the present study, incubation of Caco-2 cells with the FSs of PEWS and PEWSGM resulted in a significant upregulation of the expression levels of *occludin* gene in the pre-incubation state, compared with the untreated cells and the cells that were subjected to LPS (Figure 3). Likewise, to *zonulin-1* expression levels, the results from the other two conditions demonstrated a positive trend of fermentation products of both mushrooms to restore the impaired intestinal barrier, without, however, being significant compared to the untreated cells (Figure 3). These findings further enhance the potential effect of FSs in preventing an impending intestinal barrier dysregulation. Preventive effect of GOSs against intestinal barrier disruption was demonstrated by Akbari et al. (2015) [48]. More specifically, the authors suggested that GOSs had predominantly a preventive effect on the deoxynivalenol-induced intestinal damage in a series of in vitro models and an in vivo study in mice. Interestingly, in the same study, co-incubation of GOS for 24 h did not counteract the deoxynivalenol induced intestinal barrier disruption. More recently, Wang et al. (2021) [42] demonstrated that intragastric administration of GOS in mice attenuated intestinal barrier damage induced by LPS in the jejunum and ileum, and upregulated intestinal TJs, suggesting that GOS can act as a protective agent for intestinal health.

Claudins, along with occludins are transmembrane proteins that are important components of the TJs. Claudins are grouped into two categories known as tight claudins (claudin 1, 3, 4, 5 and 18), which control the adhesion and tightness of the intestinal barrier, and leaky claudins (claudin 2, 10 and 15) that control paracellular permeability [49]. The claudin-1 is a major component of TJs and has been proposed to have a key role for TJ integrity. In the present study, incubation of the Caco-2 cells with the FSs from PEWS mushroom resulted in a significant upregulation of *claudin-1* only in the pre-incubation condition, compared to the untreated cells and the cells that were challenged with LPS (Figure 4).

*P. eryngii*, also known as the king oyster mushroom, is widely consumed as functional food and displays multiple biological functions due to its variety of bioactive components. Among these substances, polysaccharides extracted from *P. eryngii* have been shown to exhibit antitumor, antioxidant, hepatoprotective, hypolipidemic, anti-obesity and anti-inflammatory activities [16,50,51,52,53]. A possible explanation for the preventive effect of *P. eryngii* mushrooms on impending intestinal barrier damage could be their high β-glucan content. According to previous results, the mushroom content of β-glucans was 38.7% ± 5.4 (%*w/w*) for the PEWS and 42.2% ± 5.9 (%*w/w*) for the PEWSGM [14]. Various studies have demonstrated that there is a positive association between the consumption of β-glucan-containing cereal products or cereal fibers and intestinal barrier function; however, there are limited research studies examining the β-glucans derived from edible mushrooms. According to the study of Mao et al. [54] (2019), Lentinan (LNT, deriving from *Lentinula edodes* mushrooms) administration relieved Rotavirus (RV)-induced diarrhea in piglets and improved barrier function. In another study, Lentinan exerted protective effects on the intestinal integrity and intestinal injury in a piglet model challenged with LPS [55].

Direct effects of FSs from *P. eryngii* mushrooms on the gut microbiota composition could be a reasonable explanation for the observed changes in TJs expression levels. In vitro fermentation with *P. eryngii* mushroom (PEWS) resulted in a high prebiotic index value and promoted the growth of *Bifidobacterium* spp. [14]. It is widely reported that intestinal microbiota can have profound effects on intestinal barrier, particularly on TJs repair and maintenance [56,57]. In the present study, the comparison between FSs of both mushrooms and FS-NC resulted to significant differences only in the case of FS-PEWS for the *occludin* gene in the pre-incubation state. This could be attributed to the fact that FSs originated from apparently healthy subjects, without history of gastrointestinal diseases, chronic/acute diarrhea, with good physical condition and probably a well-functioning intestinal barrier. Although many studies have attempted to elucidate the link between increased intestinal permeability and aging, especially since gastrointestinal disease burden is also increased during aging, it remains unclear whether and how aging actually causes negative effects on the intestinal barrier [58,59]. Furthermore, several studies could not establish a relationship between aging and the integrity of the intestinal barrier, while increased low-grade inflammation during aging might increase the susceptibility for gut barrier disruption [58]. Notably, incubation of Caco-2 cells with FS-NC for all three conditions led to a significant upregulation of TJs compared to Caco-2 cells that were challenged with LPS (Figure 1, Figure 2 and Figure 3), indicating that gut microbiota of volunteers could have a positive impact on restoration of TJs. The role of gut microbiota to enhance the protective effects of FSs from *P. eryngii* mushrooms is reinforced even further by evaluating the results per volunteer (Table 3).

Overall, the present study demonstrated the potential preventive effect of *P. eryngii* FSs against imminent damage to the intestinal barrier through upregulation of TJs’ genes associated with the integrity and function of the intestinal barrier. To our knowledge, this is the first study to examine the effects of FSs from edible mushrooms in terms of expression levels of TJs genes. Although this study elucidates aspects that have not been previously examined, the expression of TJs at protein level was not assessed, but it would be worthy of investigation in the future to confirm the observed preventive action of FSs.

## 5. Conclusions

In conclusion, our data highlighted the positive and potential protective effects of the rich in β-glucan *P. eryngii* mushrooms on the intestinal barrier integrity, via upregulation of TJs genes in the condition of pre-incubation, fueling further efforts to elucidate their manifold role, prebiotic included, in human health. Further studies are required to elucidate the expression and structures of TJs in order to confirm the potential beneficial action of FSs derived from edible mushrooms. This study also highlighted the role of the whole-food-based strategies (and not of specific bioactive ingredients) and the gut fermentation process in the prevention of intestinal barrier damage via potential synergistic interactions among different components of the whole foods.

## Figures and Tables

**Table 1:** Fermentation supernatants (FSs) used in the experiments.

Description	Abbreviation
FS of *P. eryngii* mushrooms cultivated in a wheat straw substrate	FS-PEWS
FS of *P. eryngii* mushrooms cultivated in a wheat straw and grape marc (ratio 1:1, *w/w*) substrate	FS-PEWSGM
FS of negative control (gut microbiota of each donor with no carbohydrate source)	FS-NC

**Table 2:** Sequences of primers used for quantitative real-time PCR.

Gene	Primer Sequences (5′-3′)	Reference
*b-actin* F	GCGCGGCTACAGCTTCA	[31]
*b-actin* R	CTTAATGTCACGCACGATTTCC	[31]
*Zonulin-1* F	TTCACGCAGTTACGAGCAAG	[32]
*Zonulin-1* R	TTGGTGTTTGAAGGCAGAGC	[32]
*Occludin* F	ACAAGCGGTTTTATCCAGAGTC	[32]
*Occludin* R	GTCATCCACAGGCGAAGTTAAT	[32]
*Claudin-1* F	TGGTCAGGCTCTCTTCACTG	[32]
*Claudin-1* R	TTGGATAGGGCCTTGGTGTT	[32]

**Table 3:** Expression levels of TJs genes under three conditions (i.e., pre-incubation, co-incubation and post-incubation) per volunteer.

**Pre-Incubation**				
		**TJs Genes**		
	**Treatment**	***zonulin-1***	***occludin***	***claudin-1***
Volunteer 1	Cells + LPS	0.508 ± 0.265	0.740 ± 0.064	0.788 ± 0.018 ^a^
	FS-NC	1.230 ± 0.085	1.325 ± 0.703	1.603 ± 0.364
	FS-PEWS	1.040 ± 0.014	1.275 ± 0.007 ^a,^*	1.200 ± 0.141
	FS-PEWSGM	1.230 ±0.057	1.245 ± 0.276	1.080 ± 0.127
Volunteer 2	Cells + LPS	0.638 ± 0.216	0.728 ± 0.219	0.540 ± 0.163
	FS-NC	0.998 ± 0.258	0.930 ± 0.240	0.905 ± 0.071
	FS-PEWS	1.180 ± 0.014 ^a^	1.025 ± 0.106	1.050 ± 0.184
	FS-PEWSGM	1.230 ± 0.014 ^a^	1.305 ± 0.035	1.085 ± 0.092
Volunteer 3	Cells + LPS	0.623 ± 0.138	0.668 ± 0.152	0.685 ± 0.170
	FS-NC	1.083 ± 0.407	0.788 ± 0.322	0.958 ± 0.463
	FS-PEWS	1.150 ± 0.113 *	1.220 ± 0.085	1.300 ± 0.014 ^a^
	FS-PEWSGM	1.060 ± 0.014	1.010 ± 0.000	1.055 ± 0.078
Volunteer 4	Cells + LPS	0.645 ± 0.035 ^a^	0.760 ± 0.071	0.800 ± 0.028
	FS-NC	1.323 ± 0.668	1.113 ± 0.392	1.008 ± 0.527
	FS-PEWS	1.820 ± 0.990	2.370 ± 0.325	2.100 ± 0.410
	FS-PEWSGM	1.025 ± 0.050	1.025 ± 0.092	0.940 ± 0.085
Volunteer 5	Cells + LPS	0.503 ± 0.103 ^†^	0.650 ± 0.014 ^a^	0.818 ± 0.095
	FS-NC	1.255 ± 0.099 *	1.105 ± 0.198	1.173 ± 0.279
	FS-PEWS	1.295 ± 0.120 *	1.900 ± 0.467	1.280 ± 0.057 *
	FS-PEWSGM	1.235 ± 0.163 *	1.150 ± 0.014 ^a^	1.850 ± 0.453
Volunteer 6	Cells + LPS	0.505 ± 0.339	0.575 ± 0.163 ^†^	0.678 ± 0.067
	FS-NC	0.770 ± 0.092	0.790 ± 0.141 *	0.993 ± 0.145
	FS-PEWS	1.010 ± 0.198	0.890 ± 0.057	0.910 ± 0.297
	FS-PEWSGM	1.010 ± 0.071 ^†^	1.020 ± 0.014	0.840 ± 0.085 *
Volunteer 7	Cells + LPS	0.560 ± 0.127	0.643 ± 0.060	0.640 ± 0.134
	FS-NC	0.985 ± 0.262	1.130 ± 0.170	1.050 ± 0.262
	FS-PEWS	2.505 ± 0.559	1.065 ± 0.035	0.790 ± 0.014 ^a^
	FS-PEWSGM	1.165 ± 0.050	0.950 ± 0.042 *	1.320 ± 0.226
Volunteer 8	Cells + LPS	0.625 ± 0.028 ^a,†^	0.698 ± 0.004 ^a^	0.743 ± 0.039
	FS-NC	0.960 ± 0.042 *	1.180 ± 0.092	1.070 ± 0.156
	FS-PEWS	1.220 ± 0.014 ^a,^*^,†^	1.605 ± 0.134	1.255 ± 0.092 *
	FS-PEWSGM	1.550 ± 0.071 *	1.510 ± 0.042 ^a,^*	1.150 ± 0.198
**Co-Incubation**				
	**Treatment**	***zonulin-1***	***occludin***	***claudin-1***
Volunteer 1	Cells + LPS	0.615 ± 0.064	0.665 ± 0.078	0.625 ± 0.064
	FS-NC	0.878 ± 0.279	0.703 ± 0.032 ^a^	0.905 ± 0.148
	FS-PEWS	0.750 ± 0.042	0.770 ± 0.099	0.675 ± 0.064
	FS-PEWSGM	1.255 ± 0.050 *	0.720 ± 0.156	1.050 ± 0.057
Volunteer 2	Cells + LPS	0.618 ± 0.018 ^a^	0.740 ± 0.000	0.603 ± 0.131
	FS-NC	0.595 ± 0.042 *	0.853 ± 0.053	0.890 ± 0.410
	FS-PEWS	0.745 ± 0.050 *	0.840 ± 0.042	0.810 ± 0.099
	FS-PEWSGM	0.760 ± 0.240	0.770 ± 0.198	0.880 ± 0.028
Volunteer 3	Cells + LPS	0.568 ± 0.244	0.518 ± 0.081	0.600 ± 0.156
	FS-NC	0.838 ± 0.315	0.745 ± 0.297	0.805 ± 0.240
	FS-PEWS	0.980 ± 0.042	1.000 ± 0.014	0.835 ± 0.106
	FS-PEWSGM	0.650 ± 0.085	0.605 ± 0.035 ^a^	0.660 ± 0.085
Volunteer 4	Cells + LPS	0.610 ± 0.042 ^a^	0.568 ± 0.117	0.633 ± 0.117 ^†^
	FS-NC	1.248 ± 0.230	0.990 ± 0.297	1.493 ± 0.194 *
	FS-PEWS	1.000 ± 0.099 **	1.250 ± 0.127 *	1.050 ± 0.071 *^,^**
	FS-PEWSGM	0.705 ± 0.120	0.510 ± 0.396	0.215 ± 0.007 ^a^
Volunteer 5	Cells + LPS	0.568 ± 0.046 ^a^	0.600 ± 0.191 ^†^	0.698 ± 0.095
	FS-NC	0.820 ± 0.410	1.058 ± 0.223 *	1.195 ± 0.332
	FS-PEWS	0.715 ± 0.064	1.545 ± 0.050 ^a^	1.610 ± 0.269
	FS-PEWSGM	0.965 ± 0.205	1.005 ± 0.078	0.840 ± 0.000
Volunteer 6	Cells + LPS	0.620 ± 0.071	0.583 ± 0.039 ^a^	0.720 ± 0.042
	FS-NC	0.645 ± 0.085	0.630 ± 0.064 **	0.738 ± 0.145
	FS-PEWS	1.585 ± 0.728	2.850 ± 2.503	2.660 ± 2.663
	FS-PEWSGM	1.040 ± 0.028 *	1.225 ± 0.007 ^a,^*	0.960 ± 0.071
Volunteer 7	Cells + LPS	0.605 ± 0.148	0.778 ± 0.152	0.680 ± 0.028 ^a,†^
	FS-NC	0.843 ± 0.004 ^a^	0.890 ± 0.113	1.043 ± 0.018 *
	FS-PEWS	0.825 ± 0.205	1.055 ± 0.276	0.935 ± 0.035
	FS-PEWSGM	0.910 ± 0.057	1.005 ± 0.007	1.045 ± 0.148
Volunteer 8	Cells + LPS	0.698 ± 0.173	0.688 ± 0.039	0.600 ± 0.028 ^a,†^
	FS-NC	0.883 ± 0.011 ^a^	0.983 ± 0.138	1.058 ± 0.018 *
	FS-PEWS	1.000 ± 0.042	1.035 ± 0.050 *	0.665 ± 0.035 ^a,^*
	FS-PEWSGM	0.945 ± 0.021	0.950 ± 0.099	0.845 ± 0.078
**Post-incubation**				
	**Treatment**	***zonulin-1***	***occludin***	***claudin-1***
Volunteer 1	Cell + LPS	0.550 ± 0.219	0.645 ± 0.064	0.710 ± 0.099
	FS-NC	1.028 ± 0.385	1.053 ± 0.336	1.110 ± 0.148
	FS-PEWS	0.890 ± 0.042	0.900 ± 0.141	0.795 ± 0.021 ^a,^**
	FS-PEWSGM	1.675 ± 0.163 *	1.290 ± 0.141	1.685 ± 0.021 ^a^
Volunteer 2	Cell + LPS	0.710 ± 0.219	0.658 ± 0.117	0.545 ± 0.445
	FS-NC	0.983 ± 0.117	0.900 ± 0.071	0.848 ± 0.124
	FS-PEWS	0.785 ± 0.064	0.930 ± 0.141	0.635 ± 0.078
	FS-PEWSGM	0.805 ± 0.064	0.715 ± 0.346	0.475 ± 0.078
Volunteer 3	Cell + LPS	0.588 ± 0.025 ^a^	0.608 ± 0.315	0.498 ± 0.216 ^†^
	FS-NC	0.733 ± 0.308	0.643 ± 0.265	0.670 ± 0.198 *
	FS-PEWS	0.670 ± 0.141	1.085 ± 0.007 ^a,^**	0.765 ± 0.177
	FS-PEWSGM	0.675 ± 0.106	0.510 ± 0.028 ^a^	0.570 ± 0.085
Volunteer 4	Cell + LPS	0.463 ± 0.194	0.483 ± 0.039 ^a^	0.608 ± 0.166
	FS-NC	1.185 ± 0.431	1.015 ± 0.078	1.183 ± 0.046
	FS-PEWS	0.690 ± 0.999	0.575 ± 0.120 ^†^	0.565 ± 0.078 **
	FS-PEWSGM	0.720 ± 0.028 ^a^	1.135 ± 0.120	0.950 ± 0.042
Volunteer 5	Cell + LPS	0.300 ± 0.156	0.425 ± 0.014 ^a^	0.440 ± 0.021 ^a^
	FS-NC	1.048 ± 0.866	0.835 ± 0.445	0.875 ± 0.629
	FS-PEWS	0.310 ± 0.014 ^a^	0.655 ± 0.035 ^a,^*	0.500 ± 0.255
	FS-PEWSGM	1.825 ± 0.318 *	1.605 ± 0.544	1.505 ± 0.530
Volunteer 6	Cell + LPS	0.670 ± 0.191	0.703 ± 0.173	0.640 ± 0.071
	FS-NC	0.968 ± 0.018	1.413 ± 0.039 ^a^	1.250 ± 0.297
	FS-PEWS	0.950 ± 0.085	1.030 ± 0.198 *	0.930 ± 0.156
	FS-PEWSGM	0.780 ± 0.226	1.075 ± 0.262	0.800 ± 0.226
Volunteer 7	Cell + LPS	0.495 ± 0.042 ^a^	0.555 ± 0.113	0.518 ± 0.053 ^a^
	FS-NC	0.895 ± 0.304	1.120 ± 0.467	0.698 ± 0.237
	FS-PEWS	1.615 ± 0.247 ^†,^**	1.467 ± 0.290 **	0.865 ± 0.219 ^†^
	FS-PEWSGM	0.750 ± 0.269	0.915 ± 0.233	0.685 ± 0.375
Volunteer 8	Cell + LPS	0.650 ± 0.163	0.543 ± 0.110	0.760 ± 0.212
	FS-NC	0.958 ± 0.124	1.110 ± 0.269	1.155 ± 0.106
	FS-PEWS	0.920 ± 0.071	1.195 ± 0.134	0.645 ± 0.092
	FS-PEWSGM	0.725 ± 0.191	0.960 ± 0.198 *	0.680 ± 0.141

Data represent the mean values ± S.D. * statistically significant compared to Cells + LPS (*p* < 0.05); ^†^ statistically significant compared to FS-NC (*p* < 0.05); ** statistically significant compared to FS-PEWSGM (*p* < 0.05); ^a^ statistically significant compared to untreated cells (*p* < 0.05).

## Data Availability

The data presented in this study are available upon request from the corresponding author.
